# Association between the degree of obstructive sleep apnea and the severity of COVID-19: An explorative retrospective cross-sectional study

**DOI:** 10.1371/journal.pone.0257483

**Published:** 2021-09-16

**Authors:** J. P. T. F. Ho, H. C. M. Donders, N. Zhou, K. Schipper, N. Su, J. de Lange

**Affiliations:** 1 Department of Oral and Maxillofacial Surgery, Amsterdam UMC and Academic Centre for Dentistry Amsterdam (ACTA), University of Amsterdam, Amsterdam, The Netherlands; 2 Department of Oral and Maxillofacial Surgery/Oral Pathology, VU University Medical Center/Academic Center for Dentistry Amsterdam (ACTA), Amsterdam, The Netherlands; 3 Department of Oral and Maxillofacial Surgery, Northwest Clinics, Alkmaar, The Netherlands; 4 Department of Oral and Maxillofacial Surgery, Isala, Zwolle, The Netherlands; 5 Department of Orofacial pain and Dysfunction, Academic Center for Dentistry Amsterdam (ACTA), University of Amsterdam and VU University Amsterdam, The Netherlands; 6 Department of Oral Public Health, Academic Center for Dentistry Amsterdam (ACTA), University of Amsterdam and Vrije Universiteit Amsterdam, Amsterdam, The Netherlands; University of Catania, ITALY

## Abstract

Obstructive sleep apnea (OSA) on its own, as well as its risk factors, have been found to be associated with the outcome of Coronavirus disease 2019 (COVID-19). However, the association between the degree of OSA and COVID-19 severity is unclear. Therefore, the aim of the study was to evaluate whether or not parameters to clinically evaluate OSA severity and the type of OSA treatment are associated with COVID-19 severity. Patient data from OSA patients diagnosed with COVID-19 were reviewed from outpatients from the Isala Hospital and patients admitted to the Isala Hospital, starting from March until December 2020. Baseline patient data, sleep study parameters, OSA treatment information and hospital admission data were collected. Apnea hypopnea index (AHI), low oxyhemoglobin desaturation (LSAT), oxygen desaturation index (ODI), respiratory disturbance index (RDI), and the type of OSA treatment were regarded as the independent variables. COVID-19 severity–based on hospital or intensive care unit (ICU) admission, the number of days of hospitalization, and number of intubation and mechanical ventilation days–were regarded as the outcome variables. Multinomial regression analysis, binary logistic regression analysis, and zero-inflated negative binomial regression analysis were used to assess the association between the parameters to clinically evaluate OSA severity and COVID-19 severity. A total of 137 patients were included. Only LSAT was found to be significantly associated with the COVID-19 severity (p<0.05) when COVID-19 severity was dichotomized as non-hospitalized or hospitalized and ICU admission or death. Therefore, our findings showed that LSAT seems to be a significant risk factor for COVID-19 severity. However, the degree of OSA–based on AHI, ODI, and RDI–and OSA treatment were not found to be risk factors for COVID-19 severity when looking at hospital or ICU admission, the number of days of hospitalization, and number of intubation and mechanical ventilation days.

## Introduction

Coronavirus disease 2019 (COVID-19) is an infectious disease, caused by the betacoronavirus now known as severe acute respiratory syndrome coronavirus 2 (SARS-CoV-2) [1, 2]. Due to the high transmission rate of this disease, it rapidly developed into a global pandemic with millions of confirmed deaths reported to the World Health Organization (WHO) [3, 4]. As vaccinations for SARS-CoV-2 successfully passed the trial phase and have been approved, they are now being administered globally. In many countries millions of people are still waiting to be vaccinated. Therefore, the strategy to apply social distancing and to use face masks still applies [5]. Another strategy that is gaining more interest, is to try to eliminate or optimize the control of risk factors for patients infected by COVID-19. Even though the symptoms and the spectrum of COVID-19 severity were found to vary between individuals, early reports showed that COVID-19 severity was more common in the presence of coexisting illnesses [6–8]. As time continues, more and more detailed evidence on the risk factors associated with COVID-19 is becoming available, such as hypertension, cardiovascular disease (CVD), diabetes, and respiratory disease [6, 9–11].

Obstructive sleep apnea (OSA) is a sleep breathing disorder in which the patient has recurrent events of upper airway collapse and obstruction. This leads to periods of absent and/or reduced respirations during sleep, also called apneas or hypopneas [12]. Recently, certain specific risk factors associated with the severity of OSA have also been reported as risk factors for COVID-19 severity, i.e., increased age, higher body mass index (BMI) and male gender [8, 13, 14]. Certain papers have postulated that not only risk factors for OSA, but OSA as a separate entity might be a risk factor for severe COVID-19 [14–18]. Cariou et al. even suggested–based on the results of the CORONADO study–that treated OSA might be associated with increased risk of death from COVID-19 [19].

However, it is still unclear to what extent the degree of OSA correlates with COVID-19 severity. Therefore, the aim of this retrospective cross-sectional study was to evaluate whether or not OSA-related parameters and the type of OSA treatment were associated with COVID-19 severity in a study population consisting solely of OSA patients diagnosed with COVID-19.

## Materials and methods

### Study approval

This retrospective study was approved by the Medical Ethics Committee, Isala Academy, Zwolle, the Netherlands (reference number 210221). Requirement for informed consent was waived. This study was done in accordance with the Declaration of Helsinki guidelines for human research, 1964, and amended in 2013 (64th World Medical Association General Assembly, Fortaleza, Brazil). It was conducted at the Department of Oral and Maxillofacial Surgery, Isala, The Netherlands and the Department of Oral and Maxillofacial Surgery of the Amsterdam University Medical Centers (UMC), location AMC, The Netherlands.

### Study population

Electronic medical records from COVID-19 patients–from outpatients from the Isala Hospital and patients admitted to the Isala Hospital (Zwolle, the Netherlands)–starting from March 1 until December 31, 2020 were accessed. After the medical records had been reviewed, patients were selected–based on inclusion and exclusion criteria–for eligibility for the study (Table 1).

**Table 1:** Inclusion criteria for the selected study population.

***Inclusion criteria***
Adults aged 18 years or older
Subjects with PSG or HSAT
Mild to severe OSA (AHI ≥5 events/h)
A positive rRT-PCR for SARS-CoV-2
***Exclusion criteria***
Absent result for AHI, LSAT, ODI, and RDI

AHI, apnea-hypopnea index; COVID-19, Coronavirus disease 2019; HSAT, home sleep apnea test; OSA, obstructive sleep apnea; LSAT, low oxyhemoglobin desaturation; ODI, oxygen desaturation index; PSG, polysomnography; rRT-PCR, RDI, respiratory disturbance index; real-time reverse transcription polymerase chain reaction; SARS-CoV-2, severe acute respiratory syndrome coronavirus 2.

Baseline patient data included gender, age, BMI, smoking, diabetes mellitus (DM), CVD (defined by the World Health Organization as a group of disorders of the heart and blood vessels e.g., coronary heart disease, cerebrovascular disease, peripheral arterial disease, and congenital heart disease), chronic obstructive pulmonary disease (COPD), chronic kidney disease, and active malignancy [20] Whenever a medical condition such as DM or CVD was not mentioned in a patient’s electronic medical record, but the corresponding medication was available (e.g., metformin and/or insulin, statins, antihypertensive drugs, and antiplatelet drug), the patient was scored positively for that disorder.

Confirmed COVID-19 was defined as a positive SARS-CoV-2 real-time reverse transcription polymerase chain reaction (rRT-PCR) on swab material, sputum or bronchoalveolar lavage samples.

### Dependent variables

COVID-19 severity was defined based on the WHO Clinical Progression Scale: (1) Mild disease: Ambulatory care; (2) Moderate disease: Hospitalized; and (3) Severe disease: Intensive care unit (ICU) admission or death [21]. A second parameter for COVID-19 severity was the number of days of hospitalization, with hospital discharge to either a nursing facility or the patient’s place of residence being used as the endpoint. If a patient was never admitted to the hospital before recovery, or died before admission, or died at home without admission, the number of days of hospitalization of the patient was 0. In addition, the number of intubation and mechanical ventilation days for the patients who were admitted in ICU or death was the third parameter for COVID-19 severity.

### Independent variables

Sleep study parameters and the type of OSA treatment were regarded as independent variables. Sleep studies consisted of in-laboratory polysomnography (PSG) or home sleep apnea test (HSAT). The sleep study parameters included apnea hypopnea index (AHI), low oxyhemoglobin desaturation (LSAT), oxygen desaturation index (ODI), and respiratory disturbance index (RDI). The degree of OSA severity was based on the degree of these 4 sleep study parameters. The sleep studies were performed and interpreted based on the latest version of the AASM Manual for the Scoring of Sleep and Associated Events: Rules, Terminology and Technical Specifications, at the time that the sleep study was conducted [22].

The type of OSA treatment was classified as either no treatment, continuous positive airway pressure (CPAP), or other–i.e., mandibular advancement device (MAD), sleep position therapy (SPT), or a combination of CPAP and/or MAD and/or SPT.

### Confounders

Gender, age, BMI, smoking, DM, CVD, COPD, chronic kidney disease, and active malignancy were regarded as the confounders.

### Statistical analysis

To assess the association between the degree of OSA and COVID-19 severity, multinomial logistic regression analysis was performed. In this analysis, ambulatory care (i.e., mild disease) was regarded as the reference category. The association between the degree of OSA and COVID-19 severity was also assessed using binary logistic regression analysis, where the COVID-19 severity was dichotomized as mild to moderate disease (ambulatory care or hospital admission) and severe disease (ICU admission or death). In the binary logistic regression analysis, ICU admission or death was regarded as the reference category. To assess the association between the degree of OSA and the number of days of hospitalization, zero-inflated negative binomial regression analysis was used because the number of days of hospitalization was count variable and contained excessive zeros [23]. Zero-inflated negative binomial models simultaneously estimate 2 separate models, i.e., logistic and count models, and the output is produced for both models respectively [23]. The logistic portion aims to assess the association between the independent variable(s) and excess zeros (0 days of hospitalization vs. >0 days of hospitalization), while the count portion aims to assess the association between the independent variable(s) and the full range of outcome scores including zero (i.e., the full range of the number of days of hospitalization in the present study) [23]. To assess the association between the degree of OSA and the number of intubation and mechanical ventilation days, zero-inflated negative binomial regression analysis was used. In such analysis, only patients who were admitted in ICU or death were included.

For each outcome, univariate analyses were first performed to assess the association between each sleep study parameter (independent variable) and the outcome. The independent variables with a p-value <0.10 were included in the subsequent multivariate analyses. For each outcome, 2 separate multivariate analyses were applied. One multivariate analysis included all the 9 confounders with the independent variables, while the other multivariate analysis included the 4 most important confounders (i.e., age, gender, BMI, and diabetes) with the independent variables. If no independent variables received a p-value of <0.10 in the univariate analysis for an outcome, the subsequent multivariate analyses were not performed. The significance level was set at 0.05. All analyses were performed using SPSS 26.0 (IBM Corp., Armonk, NY, USA) and R software 3.4.3 (R Development Core Team, Vienna, Austria) [24].

## Results

From March 1 until December 31, 2020, 1730 patients were identified in the Isala Hospital with COVID-19. Based on the inclusion and exclusion criteria, 137 patients were included in this study. Table 2 illustrates the baseline demographic characteristics of the study population. The quantitative data was found not to be normally distributed and were reported as the median with interquartile range. There were 91 males (66.4%) and 46 (33.6%) females. The median age of the study population was 68 (57–77) years and the median BMI was 31.2 (27.8–35.6) kg/m^2^. The majority of patients (69.4%) were admitted to the hospital. CVD (69.3%) was the most prevalent comorbidity, followed by COPD (34.3%) and DM (32.8%).

**Table 2:** Demographic variables of the study population.

**Patient characteristics (n = 137)**			
**Variables**	**Median (Q1-Q3)**	**Range**	**n (%)**
**Dependent variables**			
**COVID-19 severity**			
**Non-hospitalized**			42 (30.7)
**Hospitalized**			52 (38.0)
**ICU or death**			43 (31.4)
**Days of hospitalization**	4 (0–7)	0–66	
**ICU admission days**	0 (0–0)	0–60	
**Days of intubation and mechanical ventilation** **	**0 (0–0)**	**0–54**	
**Independent variables**			
**Sleep study parameters**			
**AHI**	20.3 (13.0–34.4)	5–73.7	
**LSAT**	84 (79–87)	35–99	
**ODI**	21.8 (13–35.0)	0.2–107.4	
**RDI**	22.3 (15.7–39.2)	5–130.0	
**OSA treatment**			
**None**			20 (14.6)
**CPAP**			96 (70.1)
**Other**			17 (12.4)
**Missing**			4 (2.9)
**Confounders**			
**Age**	68 (57–77)	27–96	
**Male gender**			91 (66.4)
**BMI**	31.2 (27.8–35.6)	19.5–45.8	
**Smoking**			6 (4.5) *
**DM**			45 (32.8)
**CVD**			95 (69.3)
**COPD**			47 (34.3)
**Chronic kidney disease**			21 (15.3)
**Active malignancy**			8 (5.8)

Data presented as median (Q1-Q3) or number (percentage) of patients.

* Data on 3 patients was missing;

**The median and range of the variable was only for the patients who were admitted in ICU or death.

AHI, apnea-hypopnea index; BMI, body mass index; COPD, Chronic obstructive pulmonary disease; COVID-19, Coronavirus disease 2019; CPAP, continuous positive airway pressure; CVD, cardiovascular disease; DM, diabetes mellitus; ICU, intensive care unit; LSAT, low oxyhemoglobin desaturation, ODI, oxygen desaturation index; OSA, obstructive sleep apnea; RDI, respiratory disturbance index; SD, standard deviation.

When hospitalized or ICU admission were regarded as the outcome, there was no statistically significant association between the degree of AHI, LSAT, ODI, or RDI, and COVID-19 severity in the univariate multinomial regression analyses (Table 3). The type of OSA treatment showed no significant association with COVID-19 severity as well. The multivariate multinomial logistic regression was not performed because the p-values of all the independent variables were >0.10. This indicated that neither the degree of OSA nor the type of OSA treatment being used by the patient is a risk factor for admission to hospital or to ICU due to COVID-19.

**Table 3:** Multinomial logistic regression analysis for COVID-19 severity when COVID-19 severity was categorized as non-hospitalized, hospitalized and ICU or death.

Univariate analysis								
			Hospitalized (moderate disease)				ICU or death (severe disease)	
Independent variables	Coefficient	SE	OR (95% CI)	p-value	Coefficient	SE	OR (95% CI)	p-value
**AHI**	0.003	0.012	1.003 (0.978–1.027)	0.839	0.012	0.013	1.012 (0.987–1.037)	0.348
**LSAT**	0.051	0.032	1.052 (0.989–1.119)	0.108	-0.025	0.024	0.975 (0.930–1.023)	0.306
**ODI**	-0.013	0.011	0.987 (0.966–1.008)	0.215	0.004	0.010	1.004 (0.985–1.023)	0.692
**RDI**	0.001	0.008	1.001 (0.986–1.016)	0.926	-0.002	0.008	0.998 (0.981–1.014)	0.780
**Treatment**								
**No treatment**	Ref.							
**CPAP**	-0.409	0.621	0.664 (0.197–2.243)	0.510	-0,401	0,638	0.670 (0.192–2.340)	0.670
**Other**	0.341	0.828	1.406 (0.277–7.131)	0.681	-0.336	0.918	0.714 (0.118–4.319)	0.714

p-values compare the independent variables of hospitalized COVID-19 patients with reference patients, and the independent variables of COVID-19 patients admitted to ICU or those who died with reference patients. Reference patients were COVID-19 outpatients from the Isala Hospital. p<0.05 was considered statistically significant.

The multivariate analysis was not conducted because the p-values of all the independent variables were <0.10 in the univariate analyses.

AHI, apnea-hypopnea index; CPAP, continuous positive airway pressure; ICU, intensive care unit; LSAT, low oxyhemoglobin desaturation, ODI, oxygen desaturation index; OR, odds ratio; RDI, respiratory disturbance index, SE; standard error.

**Fig 1:**
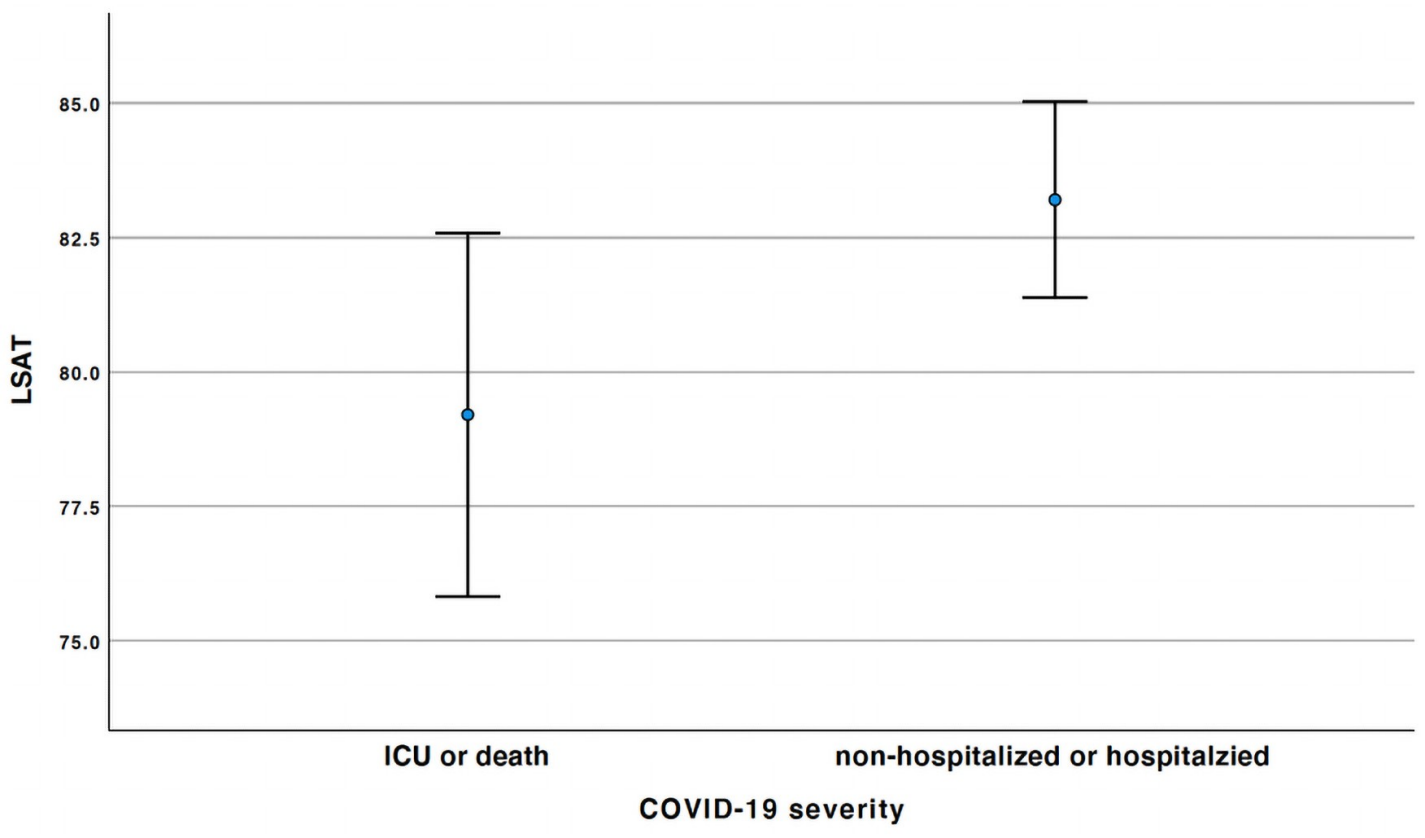
The means and 95% confidence intervals of the LSAT between the patients who were non-hospitalized or hospitalized and those who were admitted at ICU or death. Error bar graph, where the X-axis illustrates COVID-19 severity dichotomized as non-hospitalized or hospitalized and ICU or death. The Y-axis illustrates mean LSAT. COVID-19, Coronavirus disease 2019; ICU, intensive care unit; LSAT, low oxyhemoglobin desaturation.

**Fig 2:**
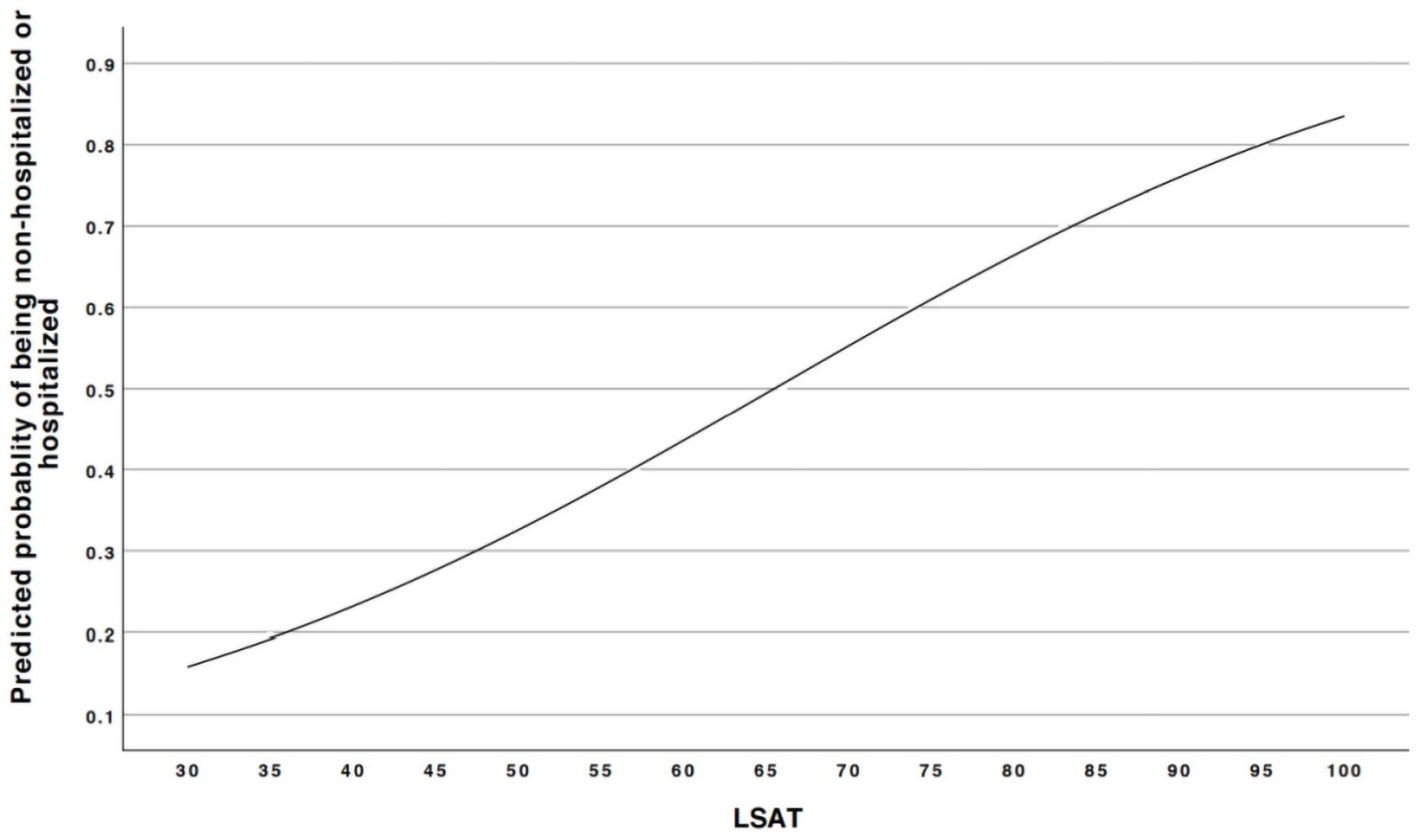
The predicted probabilities of being non-hospitalized or hospitalized of patients based on the LSAT. Prediction probability graph, where the X-axis illustrates mean LSAT. Y-axis illustrates the predicted probability for being non-hospitalized or hospitalized. LSAT, low oxyhemoglobin desaturation.

Only LSAT was found to be significantly associated with COVID-19 severity (p = 0.037) in a univariate binary logistical analysis when COVID-19 severity was dichotomized. Therefore, LSAT was included in the subsequent multivariate binary logistic regression analysis. After adjusting for all 9 confounders, LAST was still found to be significantly associated with COVID-19 severity (p = 0.035) (Table 4). When LSAT increases by one unit, patients have 1.064 times higher odds to be non-hospitalized or hospitalized than having ICU admission or death (Figs 1 and 2).

**Table 4:** Binary logistic regression analysis for COVID-19 severity when COVID-19 severity was dichotomized as non-hospitalized or hospitalized and ICU or death.

	Univariate analysis				Multivariate analysis (adjusted for all the 9 confounders: age, gender, BMI, smoking, DM, CVD, COPD, Chronic kidney disease, active malignancy)				Multivariate analysis (adjusted for all the 9 confounders: age, gender, BMI, smoking, DM, CVD, COPD, Chronic kidney disease, active malignancy)			
	Non-hospitalized/hospitalized vs. ICU or death (reference category)				Non-hospitalized/hospitalized vs. ICU or death (reference category)				Non-hospitalized/hospitalized vs. ICU or death (reference category)			
Independent variables	Coefficient	SE	OR (95%CI)	P-value	Coefficient	SE	OR (95%CI)	P-value	Coefficient	SE	OR (95%CI)	P-value
**AHI**	-0.010	0.010	0.990 (0.970–1.010)	0.318								
**LSAT**	0.047	0.023	1.048 (1.003–1.096)	0.037	0.062	0.029	1.064 (1.004–1.127)	0.035	0.059	0.027	1.061 (1.007–1.119)	0.027
**ODI**	-0.011	0.008	0.989 (0.973–1.006)	0.197								
**RDI**	0.003	0.007	1.003 (0.989–1.017)	0.697								
**Treatment**												
**No treatment**	Ref.											
**CPAP**	0.169	0.518	1.185 (0.429–3.269)	0.744								
**Other**	0.560	0.739	1.750 (0.411–7.454)	0.449								

p values for the univariate analysis compare the independent variables and the COVID-19 severity: non-hospitalized or hospitalized vs. ICU or death (reference category. p< 0.05 is considered statistically significant.

AHI, apnea-hypopnea index; BMI, body mass index; CI, confidence interval; COPD, Chronic obstructive pulmonary disease; COVID-19, Coronavirus disease 2019; CPAP, continuous positive airway pressure; CVD, cardiovascular disease; DM, diabetes mellitus; ICU, intensive care unit; LSAT, low oxyhemoglobin desaturation, ODI, oxygen desaturation index; OR, odd ratio; OSA, obstructive sleep apnea; RDI, respiratory disturbance index; RR, risk ratio; SE, standard error.

When the number of days of hospitalization was regarded as the outcome, there was no statistically significant association between the degree of the AHI, LSAT, ODI, RDI, or treatment and the number of days of hospitalization for either the logistic model or the count model in the univariate zero-inflated negative binomial regression analyses (Table 5). However, because the p-value of OSA treatment in the count model was <0.10 when CPAP was compared with no treatment, OSA treatment was included in the subsequent multivariate analysis. The multivariate analysis adjusting for all 9 confounders showed that there was no significant association between the type of OSA treatment and the number of days of hospitalization. When adjusted for age, gender, BMI, and DM only, no significant association was found between the type of OSA treatment and the number of days of hospitalization. This indicated that neither the degree of OSA nor the type of OSA treatment being used by the patient is a risk factor for the days of hospitalization due to COVID-19.

**Table 5:** Zero-inflated negative binomial regression analysis for the number of days of hospitalization.

	Univariate analysis								Multivariate analysis (adjusted for all confounders: age, gender, BMI, smoking, DM, CVD, COPD, chronic kidney disease, active malignancy)								Multivariate analysis (adjusted for age, gender, BMI, DM only)							
	Logistic model				Count model				Logistic model				Count model				Logistic model				Count model			
Independent variables	Coefficient	SE	OR (95% CI)	P-value	Coefficient	SE	RR (95% CI)	P-value	Coefficient	SE	OR (95% CI)	P-value	Coefficient	SE	RR (95% CI)	P-value	Coefficient	SE	OR (95% CI)	P-value	Coefficient	SE	RR (95% CI)	P-value
**AHI**	-0.014	0.015	0.986 (0.957–1.015)	0.348	0.001	0.006	1.001(0.989–1.013)	0.836																
**LSAT**	-0.020	0.024	0.980 (0.933–1.027)	0.392	-0.015	0.015	0.985 (0.956–1.015)	0.328																
**ODI**	0.001	0.011	1.001 (0,979–1.023)	0.947	0.002	0.005	1.002 (0.992–1.012)	0.758																
**RDI**	-0.008	0.010	0.992 (0.972–1.012)	0.456	-0.006	0.004	0.994 (0.986–1.002)	0.147																
**Treatment**																								
**No treatment**	Ref.																							
**CPAP**	0.386	0.756	1.471 (-0.011–2.953)	0.610	0.610	0.318	1.840 (1.218–2.463)	0.055	-0.838	0.757	0.433 (-1.051–1.916)	0.268	0.480	0.312	1.616 (1.005–2.228)	0.124	-0.792	0.732	0.453 (-0.982–1.888)	0.279	0.306	0.311	0.358 (0.748–1.968)	0.325
**Other**	-0.398	1.170	0.672 (-1.622–2.965)	0.734	0.000	0.418	1.000 (0.181–1.819)	1.000	-0.145	1.051	0.865 (-1.195–2.925)	0.890	0.068	0.380	1.070 (0.326–1.815)	0.859	-0.420	1.021	0.657 (-1.344–2.658)	0.681	-0.177	0.384	0.838 (0.085–1.590)	0.645

p values for the logistic portion compare the independent variables and excess zeros. p values for the count portion compare the independent variables and the full range of outcome scores, including zero. p< 0.05 is considered statistically significant.

AHI, apnea-hypopnea index; BMI, body mass index; CI, confidence interval; COPD, Chronic obstructive pulmonary disease; COVID-19, Coronavirus disease 2019; CPAP, continuous positive airway pressure; CVD, cardiovascular disease; DM, diabetes mellitus; ICU, intensive care unit; LSAT, low oxyhemoglobin desaturation, ODI, oxygen desaturation index; OR, odd ratio; OSA, obstructive sleep apnea; RDI, respiratory disturbance index; RR, risk ratio; SE, standard error.

When the number of intubation and mechanical ventilation days was regarded as the outcome, only LSAT was found to be significantly associated with COVID-19 severity (p = 0.04) for the count model in the univariate zero-inflated negative binomial regression analyses for patients who were admitted in ICU or death (Table 6). This indicates that when LSAT increases, patients may have lower number of intubation and mechanical ventilation days. However, in the logistic portion of the model, LSAT is not significantly associated with the presence or absence of intubation and mechanical ventilation (P = 0.77).

**Table 6:** Zero-inflated negative binomial regression analysis for the number of intubation and mechanical ventilation days for patients who were admitted in ICU or death.

	Univariate analysis								Multivariate analysis (adjusted for all confounders: age, gender, BMI, smoking, DM, CVD, COPD, chronic kidney disease, active malignancy)								Multivariate analysis (adjusted for age, gender, BMI, DM only)							
	Logistic model				Count model				Logistic model				Count model				Logistic model				Count model			
Independent variables	Coefficient	SE	OR (95% CI)	P-value	Coefficient	SE	RR (95% CI)	P-value	Coefficient	SE	OR (95% CI)	P-value	Coefficient	SE	RR (95% CI)	P-value	Coefficient	SE	OR (95% CI)	P-value	Coefficient	SE	RR (95% CI)	P-value
**AHI**	-0.002	0.013	0.998 (0.973–1.023)	0.863	0.013	0.009	1.013 (0.995–1.031)	0.150																
**LSAT**	0.012	0.043	1.012 (0.928–1.096)	0.770	-0.045	0.022	0.956 (0.913–0.999)	0.041	NA	NA	NA	NA	NA	NA	NA	NA	-0.035	0.052	0.966 (.,864–1.068)	0.504	-0.015	0.016	0.985 (0.954–1.016)	0.337
**ODI**	0.000*	0.014	1.000 (0.973–1.027)	0.998	0.012	0.008	1.012 (0.996–1.028)	0.112																
**RDI**	0.004	0.013	1.004 (0.979–1.029)	0.740	0.005	0.01	1.005 (0.985–1.025)	0.625																
**Treatment**																								
**No treatment**	NA	NA	NA	NA	NA	NA	NA	NA																
**CPAP**	NA	NA	NA	NA	NA	NA	NA	NA																
**Other**	NA	NA	NA	NA	NA	NA	NA	NA																

*: the exact coefficient was 0.0000332

AHI, apnea-hypopnea index; BMI, body mass index; CI, confidence interval; COPD, Chronic obstructive pulmonary disease; COVID-19, Coronavirus disease 2019; CPAP, continuous positive airway pressure; CVD, cardiovascular disease; DM, diabetes mellitus; ICU, intensive care unit; LSAT, low oxyhemoglobin desaturation, NA, not able to analyze due to insufficient sample size; ODI, oxygen desaturation index; OR, odd ratio; OSA, obstructive sleep apnea; RDI, respiratory disturbance index; RR, risk ratio; SE, standard error.

Because in the variable “treatment”, the categories “no treatment” (N = 7) and “other” (N = 4) had very small sample size, it is insufficient to conduct the regression analysis.

The multivariate analysis adjusting for all 9 was not able to be conducted, due to the small sample size. When adjusted for age, gender, BMI, and DM only, for both the count and logistic model portion, no significant association was found between LSAT and the number of intubation and mechanical ventilation days (P = 0.34; P = 0.50).

## Discussion

Although there is enormous interest in everything related to COVID-19, a lot is still unclear about the association between OSA and COVID-19. The present study therefore examined the degree of OSA as a risk factor for COVID-19 severity in a study population consisting solely of OSA patients diagnosed with COVID-19.

Strausz et al. found that OSA is associated to COVID-19 severity, determined on non-admission or admission to hospital–which is similar to one of the dependent variables of the definition of COVID-19 severity used in our study [18]. In order study if the degree of OSA is associated to COVID-19 severity, the present study used sleep study parameters, specifically AHI, LSAT, ODI, and RDI, to see whether or not there was an association with ambulatory care (mild disease), hospital admission (moderate disease) and/or ICU admission (severe disease). LSAT showed to have a significant association with the COVID-19 severity. Mean LSAT is higher in patients who are hospitalized or non-hospitalized than those with ICU or death (Fig 1). Additionally, when LSAT is increasing, patients are more likely to be non-hospitalized or hospitalized than having ICU admission or death (Fig 2). A possible explanation for the association between LSAT and COVID-19 severity could be that LSAT is decreased not only based on upper airway obstruction during sleep in OSA patients, but also due to disease-related gas exchange deficits e.g., ventilation and perfusion mismatch. Lower baseline oxygen saturation measured by pulse oximeter and oxygen saturation measured by blood analysis have also been reported to be a risk factor for COVID-19 severity based on gas exchange deficits [25, 26]. Messineo et al., found that greater breath-hold induced desaturation–indicative for gas exchange deficit–was associated with COVID-19 severity [26]. Secondly, an inadequate ventilatory control, in the case of silent hypoxia has also been reported to be a risk factor for COVID-19 severity [27]. Likewise, patients with OSA could be predisposed to have a more tolerable lower oxygen saturation level without dyspnea, until they are infected with SARS-CoV-2. Messineo et al., also reported that inadequate ventilatory control tested with greater breath-holding tolerance was associated with COVID-19 severity [26]. The results showed that the degree of OSA–based on AHI, ODI, and RDI–has no significant association with COVID-19 severity.

In contrast to the study by Strausz et al., the present study also investigated whether the degree of OSA is associated with COVID-19 severity based on the number of days of hospitalization, and the number of intubation and mechanical ventilation days. The results showed that the degree of OSA is not significantly associated with the number of days of hospitalization nor the number of intubation and mechanical ventilation days.

A recently published systematic review on COVID-19 and OSA by Miller and Cappuccio concluded that there might be an association between certain risk factors and comorbidities associated with OSA, which are also associated with poor COVID-19 outcomes [28]. However, this systematic review included only 6 studies containing original data, of which 1 was a case study and 4 were case series with a maximum sample size of fewer than 10 OSA patients [29–33]. In addition, 1 included study was the CORONADO study by Cariou et al., which was a large national observational study not primarily consisting of OSA patients [19]. Furthermore, this literature search was performed up until June 2, 2020. Since then, there has been a number of publications on this topic. Hence, there is a need to undertake a more up-to-date and sufficiently rigorous synthesis of the fast-growing body of evidence in future.

Maas et al. published a study in which they found that OSA was associated with an increased risk of hospitalization and respiratory failure [17]. Cade et al. published their results in a letter to the editor, in which they concluded that OSA is a risk factor for COVID-19 mortality [16]. Strausz et al. concluded that, based on their study results, OSA was associated with higher risk for hospitalization [18]. However, one should be careful with interpreting the results of the studies by Cariou et al., Maas et al., Cade et al., and Strausz et al. These studies were all large patient registry observational studies in which the presence of the diagnosis of OSA was evaluated [16–19]. This can introduce flawed coding of administrative data, which might result in imprecise study results. In addition, one should note that these studies only registered the presence of OSA as a risk factor for COVID-19 severity, but they did not take into account the degree of OSA.

Our study results show that there is no association between the type of OSA treatment and COVID-19 severity. This is in contrast to the CORONADO study, in which results suggested that treated OSA might be associated with increased risk of death from COVID-19 [19]. However, caution should be exercised due to the fact that the authors were not able to evaluate to what extent the patients in the present study population were effectively treated for their OSA. This issue was also not addressed in the paper on the CORONADO study.

One should be mindful when interpreting the presented results and conclusions. The present study showed that 150 of 1730 (7.9%) patients were COVID-19 positive and had a history of OSA. This is similar to the 38 out of 445 (8.5%) patients found by Strausz et al. [18]. However, this is a lower prevalence compared to other studies, ranging up to almost 30% of patients [30–34]. This might explain why the outcomes of the present study differ from those of other studies. In retrospective cross-sectional studies a certain amount of bias could be introduced due to a number of confounders and the lack of available data, i.e., COVID-19 patients with undiagnosed OSA, the degree of patient compliance and adherence to OSA therapy, and, as previously discussed, the effectiveness of the OSA therapy. In addition, the severity of COVID-19 was based on hospital and/or ICU admission, the number of days of hospitalization, and number of intubation and mechanical ventilation days. One could argue that more quantifiable physiological and blood test parameters might have expressed COVID-19 severity more adequately, i.e., temperature, blood pressure, heart rate, hemoglobin, thrombocytes, leukocytes, creatinine, lymphocytopenia, C-reactive protein, and procalcitonin [34]. No sleep study data was available from the time the patient was diagnosed with COVID-19. Older and more recent sleep study data were all added to the study data, with no limitation for maximal range for sleep study data. This might introduce some bias. In addition, it would have been interesting to see what effect COVID-19 had on the patient’s OSA with regard to the sleep study parameters. Furthermore, hypoxic burden in contrast to AHI has been suggested to be able to predicts CVD mortality across populations [35]. This OSA severity metric does not only take in to account the number of apneic events, but also the hypoxemia by measuring the respiratory event-associated area under the desaturation curve from pre-event baseline [35, 36]. One might raise the question if the degree of hypoxic burden is associated with COVID-19 severity. Based on the available data, regrettably this was not an issue that was able to be investigated.

While taking all these issues into account, the authors still believe that the results of this study can provide good insight into the association between the degree of OSA and COVID-19 severity. Even though the severity of COVID-19 can vary between individuals, the disease can have a detrimental outcome, and as the pandemic continues, more and more hospital resources are being depleted. In order to optimize patient care and hospital resources, it is important to identify and also exclude risk factors, since this can be beneficial for at-risk and high-risk individuals.

To gain more insight into the association between OSA and COVID-19, the authors advocate for more research with optimal data, i.e., larger study populations pertaining specifically to OSA and COVID-19 patients, adequate sleep study data prior to but also after COVID-19 diagnosis, physiological and blood test parameters, and available data on patient compliance with and adherence to OSA therapy.

## Conclusions

The study findings showed that there was no significant association between AHI, ODI, or RDI and COVID-19 severity, while LSAT was found to be significantly associated with COVID-19 severity when looking at hospital or ICU admission. There was no significant association between AHI, ODI, RDI, or LSAT and the number of days of hospitalization. COVID-19 severity is also not significantly associated with a specific type of OSA treatment. Therefore, the degree of OSA–based on AHI, ODI or RDI–and the type of OSA treatment are not found to be risk factors for COVID-19 outcome, while LSAT seemed to be a significant risk factor for COVID-19 severity.

## Supporting information

S1 Table Demographic variables of the study population for COVID-19 severity. Data presented as median (Q1-Q3) or number (percentage) of patients. * Data on 3 patients was missing. AHI, apnea-hypopnea index; BMI, body mass index; COPD, Chronic obstructive pulmonary disease; COVID-19, Coronavirus disease 2019; CPAP, continuous positive airway pressure; CVD, cardiovascular disease; DM, diabetes mellitus; ICU, intensive care unit; LSAT, low oxyhemoglobin desaturation, ODI, oxygen desaturation index; OSA, obstructive sleep apnea; RDI, respiratory disturbance index; SD, standard deviation.
(DOCX)
