# Supplementary material for: Association between the degree of obstructive sleep apnea and the severity of COVID-19: An explorative retrospective cross-sectional study
Source: PLoS One. 2021 Sep 16;16(9):e0257483. doi: 10.1371/journal.pone.0257483 (PMC8445416; doi:10.1371/journal.pone.0257483)
Supplement: S1 Table — Data presented as median (Q1-Q3) or number (percentage) of patients. * Data on 3 patients was missing. AHI, apnea-hypopnea index; BMI, body mass index; COPD, Chronic obstructive pulmonary disease; COVID-19, Coronavirus disease 2019; CPAP, continuous positive airway pressure; CVD, cardiovascular disease; DM, diabetes mellitus; ICU, intensive care unit; LSAT, low oxyhemoglobin desaturation, ODI, oxygen desaturation index; OSA, obstructive sleep apnea; RDI, respiratory disturbance index; SD, standard deviation. (DOCX) [file pone.0257483.s001.docx]

|  | **Non-hospitalized (n=42)** | | | **Hospitalized (n=52)** | | | **ICU or death (n=43)** | | |
| --- | --- | --- | --- | --- | --- | --- | --- | --- | --- |
|  | **Median (Q1-Q3)** | **Range** | **n (%)** | **Median (Q1-Q3)** | **Range** | **n (%)** | **Median (Q1-Q3)** | **Range** | **n (%)** |
| **Independent variables** | | | | | | | | | |
| **Sleep study parameters** | | | | | | | | | |
| AHI | 19.5 (12.4-32.7) | 5-66 |  | 19.2 (12.1-33.7) | 5.7-73.7 |  | 21.6 (14.2-35.6) | 6.4-71.2 |  |
| LSAT | 84 (79.3-87.5) | 35-99 |  | 85 (81-88) | 70-99 |  | 82 (75-86) | 40-92 |  |
| ODI | 23.2 (15.1-34.2) | 4.8-99 |  | 20.5 (8.0-34.8) | 0.2-71.9 |  | 24.2 (13.5-40.3) | 3-107.4 |  |
| RDI | 21.1 (15.9-46.7) | 5-109.3 |  | 25.5 (15.3-46.0) | 6.5-99 |  | 22.3 (15-28.6) | 6.4-130.0 |  |
|  | | | | | | | | | |
| **OSA treatment** | | | | | | | | | |
| None |  |  | 5 (11.9) |  |  | 8 (15.4) |  | 7 (16.3) |  |
| CPAP |  |  | 32 (76.2) |  |  | 34 (65.4) |  | 30 (70.0) |  |
| Other |  |  | 4 (9.5) |  |  | 9 (17.3) |  | 4 (9.3) |  |
|  | | | | | | | | | |
| **Confounders** | | | | | | | | | |
| Age | 63 (48.8-75) | 27-96 |  | 63.5 (54.3-72.8) | 33-85 |  | 70 (66-78) | 51-88 |  |
| Male gender |  |  | 21 (50) |  |  | 35 (67.3) |  |  | 35 (81.4) |
| BMI | 36.3 (33.6-41.0) | 32.0-45.8 |  | 27.9 (25.0-30.1) | 19.5-40.8 |  | 31.1 (27.9-32.6) | 20.3-42.6 |  |
| Smoking |  |  | 2 (4.7) |  |  | 3 (6) # |  |  | 1 (2.4) * |
| DM |  |  | 16 (38.1) |  |  | 13 (25) |  |  | 16 (37.2) |
| CVD |  |  | 32 (76.2) |  |  | 36 (69.2) |  |  | 27 (62.8) |
| COPD |  |  | 15 (35.7) |  |  | 18 (34.6) |  |  | 14 (32.6) |
| Chronic kidney disease |  |  | 7 (16.7) |  |  | 8 (15.4) |  |  | 6 (14.0) |
| Active malignancy |  |  | 1 (2.4) |  |  | 1 (2.0) |  |  | 6 (14.0) |

**S1 Table. Demographic variables of the study population for COVID-19 severity**

Data presented as median (Q1-Q3) or number (percentage) of patients.

* Data on 3 patients was missing.

AHI, apnea-hypopnea index; BMI, body mass index; COPD, Chronic obstructive pulmonary disease; COVID-19, Coronavirus disease 2019; CPAP, continuous positive airway pressure; CVD, cardiovascular disease; DM, diabetes mellitus; ICU, intensive care unit; LSAT, low oxyhemoglobin desaturation, ODI, oxygen desaturation index; OSA, obstructive sleep apnea; RDI, respiratory disturbance index; SD, standard deviation.
